# Association of gait and cognition after surgery in patients with idiopathic normal pressure hydrocephalus

**DOI:** 10.1038/s41598-023-45629-8

**Published:** 2023-10-27

**Authors:** Masatsune Ishikawa, Etsuro Mori

**Affiliations:** 1 Rakuwa Villa Ilios, Kyoto, Kyoto Japan; 2 Normal Pressure Hydrocephalus Center, Otowa Hospital, Kyoto, Kyoto Japan; 3 Department of Behavioral Neurology and Neuropsychiatry, Osaka University United Graduate, Toyonaka, Osaka Japan

**Keywords:** Neuroscience, Neurology

## Abstract

Idiopathic normal pressure hydrocephalus (iNPH) is a treatable disease in older adults. The association between gait and cognition has recently become a topic of interest. Sequential changes in this association were investigated in patients with iNPH using a newly developed statistical method. Data were extracted from the SINPHONI-2 multicenter study on iNPH. Fifty patients who underwent shunt surgery were included in this study. Gait and cognition were assessed using the Timed Up and Go (TUG) and Mini-Mental State Examination (MMSE) tests. In addition to the MMSE total score, changes in the sub-item scores were examined. The ordinal sub-items of the MMSE are usually treated as continuous or categorical; however, both are unsuitable. An ordinal smoothing penalty with a generalized additive model enables precise statistical inference of ordinal and binary predictors. The TUG time improved significantly at 3, 6, and 12 months after surgery. The MMSE total scores increased without statistical significance. Preoperatively, there was no association between TUG time and MMSE sub-items. At 3 months, the “Registration,” ”3-step command,” “Read,” and “Copy” sub-items were statistically significant. The number of significant sub-items increased after 12 months. Thus, the association between gait and cognition gradually increased after surgery in patients with iNPH.

## Introduction

Idiopathic normal pressure hydrocephalus (iNPH) is a treatable disease that affects gait, cognition, and continence in older adults, with symptomatic improvement after cerebrospinal fluid (CSF) shunt surgery^1,2^. Gait disturbance is the main symptom of iNPH and is characterized by a short-stepped, wide-based gait with or without hesitation. CSF shunt surgery can reverse gait disturbances during the early postoperative period. In contrast, although iNPH is a well-known treatable form of dementia^1^, the improvement in dementia after surgery is often not as remarkable as that of gait disturbance^3^.

The Mini-Mental-State Examination (MMSE) is a widely used measure of global cognitive impairment. MMSE examines cognitive functions in five domains: orientation, memory, attention, language, and visual construction. The total MMSE score is the sum of the sub-item scores, with a maximum score of 30. Several studies have been conducted using the MMSE sub-items. In patients with Alzheimer's disease, the sub-item “Copy” had an important effect on the decrease in basic and instrumental activities of daily living (ADL)^4^. Another study showed that the “Time” and “Place” sub-items of the MMSE showed a significant association with most sub-items of the Physical Self-Maintenance Scale and instrumental ADL in Alzheimer’s disease^5^. Older drivers who had difficulty with the “Place” sub-item were more than six times more likely to be involved in a future crash^6^.

The MMSE sub-items were ordinal and binary variables. Mathematically, ordinal variables cannot be added or subtracted, although the MMSE total score was originally designed as the summed score of all sub-items. Previous studies have treated MMSE sub-items as continuous or categorical. Both are unsuitable, as continuous disregards the discrete nature, while categorical disregards the ordinal nature. The recent development of the ordinal smoothing penalty has increased the reliability of ordinal predictor assessments^7,8^. Furthermore, the combined use of the generalized additive model (GAM)^7^ allows statistical inference of ordinal and binary predictors.

The association between gait and cognition in older people has recently been well recognized^9^. In patients with iNPH, cognitive impairment is closely associated with gait disturbances^10^. However, the gait-cognition relationship in patients with iNPH after shunt surgery is unknown. In this study, we investigated whether gait improvement after shunt surgery in patients with iNPH is associated with cognitive changes and which cognitive sub-items are closely related to gait improvement, using data from the SINPHONI-2 multicenter cooperative study of iNPH in Japan^11,12^. Gait was assessed using the 3-m Timed Up and Go (TUG) test. Changes in cognition were assessed using MMSE sub-items. As the sub-items were ordinal or binary, the association between gait and cognition was investigated in patients with iNPH using a newly developed statistical method of ordinal smoothing penalty with a GAM.

## Methods

### Participants

**Figure 1:**
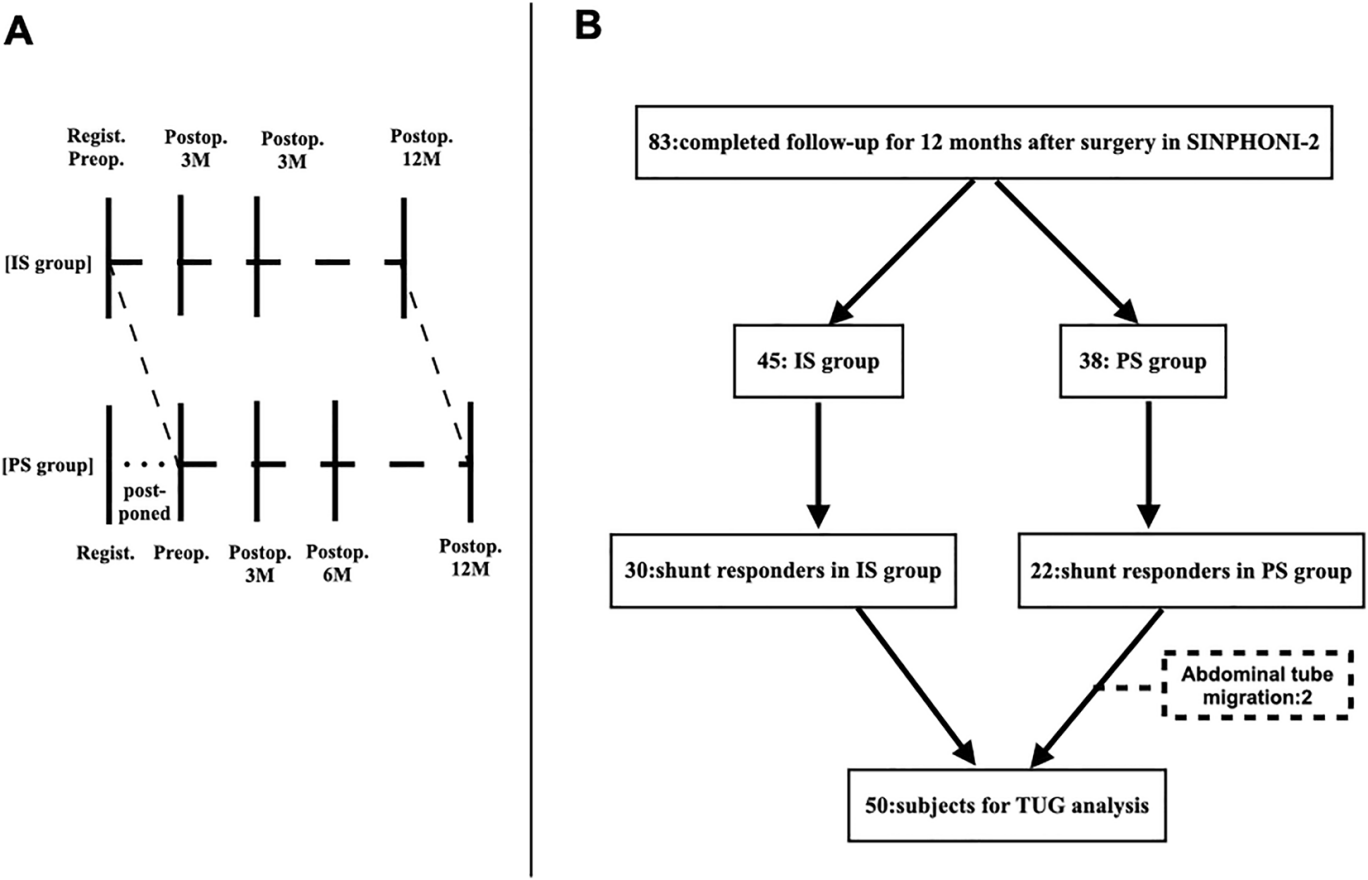
(**A**) Study design of SINPHONI-2. In the PS group, surgery is postponed for 3-months. Both the IS and PS groups are followed up for 12 months after surgery. *IS* immediate surgery, *PS* postponed surgery, *Regist.* Registration, *3M, 6M, 12M*, 3, 6, and 12 months, *SINPHONI-2* study of idiopathic normal pressure hydrocephalus in neurological impairment-second study. (**B**) Flowchart for patient selection. Shunt responders are defined as those with improvements in ADL of one or more grades on the mRS from the preoperative state. Missing data are observed in two patients during follow-up. Therefore, a total of 50 patients were included in this study. TUG, Timed Up and Go test.

SINPHONI-2 is an open-label randomized trial (UMIN-CTR: UMIN000002730, first registration: 01/02/2010) that followed the Guidelines for Good Clinical Practice and adhered to the principles of the Declaration of Helsinki (2002) of the World Medical Association. The study protocol was approved by the Tohoku University Hospital Ethics Committee and the Institutional Ethics Committee at each site. Written informed consent was obtained from all patients or their representatives. All clinical and radiological data were prospectively recorded in an independent protocol compliance center using a web-based case report system. Details of the participants, definitions of iNPH, protocol compliance, and data collection (including data acquisition and management) have previously been described. Briefly, 102 candidates diagnosed with possible iNPH according to the second edition of the Japanese iNPH guidelines^13^ were recruited from 20 Japanese centers between March 2010 and October 2011. The inclusion criteria for this study were age 60–85 years at entry, presence of one or more symptoms (e.g., gait disturbance, cognitive impairment, and urinary disturbance) based on the iNPH grading scale within 3 months before the provision of consent, ventriculomegaly with an Evans index of > 0.3, concurrent narrow sulci at high convexity, and an enlarged Sylvian fissure observed on computed tomography or magnetic resonance imaging. The exclusion criteria were the presence of secondary or congenital hydrocephalus or aqueductal stenosis, CSF pressure ≥ 20 cmH_2_O, complications of severe disuse muscle atrophy, and psychiatric disorders or other neurological diseases. According to these criteria, 93 patients were included and randomly assigned to the immediate surgery (IS) group or the 3-month-postponed surgery (PS) group (Fig. 1A). Randomization (1:1 ratio) was performed using a permuted block design with a block size of four or six within each clinical center according to a randomized code generated by the trial statistician. All patients in the IS group underwent lumboperitoneal shunt surgery using a Codman–Hakim programmable valve with a SiphonGuard (Codman Neuro-DePuy Synthes, Raynham, MA, USA). In the PS group, all patients underwent lumboperitoneal shunt surgery 3 months after registration. During the 3 months, patients in the PS group were instructed to perform physical tasks.

### Assessment of gait and cognition

In SINPHONI-2, gait performance was assessed in seconds using a 3-m TUG test^14^, which measures the time required to complete the six components of standing up from a chair, walking 3 m, turning around 180°, walking back to the chair, turning around 180° again, and sitting back down on the chair. All tests were performed twice. As patients with severe conditions occasionally experienced interruptions in the second trial, data from the first trial were used. Global cognition was assessed using the MMSE, which assesses five cognitive domains: orientation, memory, attention, language, and visual construction. The MMSE is composed of 11 sub-items, with total points ranging from 0 to 30^15^: orientation to time and place (5 points each), immediate memory registration (3 points), attention and calculation (5 points, serial 7 subtractions), recall (3 points), naming (2 points), repeating (1 point), 3-step command (3 points), reading and obeying (1 point), writing (1 point), and copying (1 point). In SINPHONI-2, the Japanese version of the MMSE was used^16^. Additionally, the total score of the Frontal Assessment Battery (FAB)^17^ was used to assess executive function.

### Selection of participants in this study

Among the 102 potential participants, nine were eliminated due to deviations from the inclusion criteria. Among the 93 patients in this study, 83 completed 12-month-follow-up after surgery (45 in the IS group and 38 in the PS group). Shunt responders were defined as those with one or more improvements 12 months after surgery compared with the preoperative modified Rankin scale (mRS) score. There were 30 shunt responders in the IS group and 22 shunt responders in the PS group. In the PS group, preoperative data on MMSE sub-items were not obtained in two patients. The patient underwent abdominal tube migration and required shunt revision. Subsequently, the patient did not visit the clinic. Therefore, 50 patients were included in the study of the relationship between TUG time and MMSE scores (Fig. 1B).

### Statistical analysis

Among the SINPHONI-2 variables, age, sex, education (years), TUG time (s), mRS scores (0–6), iNPH grading scale (0–4), MMSE total and sub-item scores, and FAB total score were evaluated. The TUG time, MMSE total score, and FAB total score were continuous variables. The mRS and iNPH grading scales were considered ordinal variables. MMSE sub-item scores of three or more levels were considered ordinal variables, and sub-items of two levels were considered binary variables. These data were assessed at four different time points (at registration and 3, 6, and 12 months after surgery). TUG data were not obtained for two patients at 3 months, three at 6 months, and two at 12 months. All statistical analyses were performed with R software^18^. Continuous data were regarded as normally distributed according to the central limit theorem, which states that the sampling distribution of the mean tends towards a normal distribution, even if the original variables are not normally distributed^19^. Statistical significance was set at p < 0.05. The mean and standard error values for continuous variables were compared using parametric *t*-tests, and categorical data were analyzed using the Chi-square test. Group comparisons with ordinal data were performed using Spearman’s rank-sum test. Continuous data were plotted with means and standard errors using the “sciplot” package^20^.

The MMSE sub-items are ordinal and binary. A smoothing spline approach to the modeling of ordinal predictors is useful^21^, and the “ordPens” R package^7^ is the only package currently available for this purpose^22^. Smoothing of the ordinal variables of the sub-items was implemented using the “ordSmooth” function in the “ordPens” package^7^, applying the smoothing penalty for adjacent dummy coefficients. The smoothed data were then analyzed with GAM, using the “gam” function in the “mgcv” R package^23^. Binary data were treated as continuous in the “gam” function. Since the major interest was the relationship between TUG time and each sub-item, a univariate regression analysis for the respective sub-items was performed. GAM can draw inferences about associations between outcomes and predictors without placing parametric restrictions on the association^24^. This allows analyses for ordinal and binary data using the same statistical method. The p-values for ordinal data were reported only in the case of a second-order penalty, which was reliable^8^.

### Ethics declarations

SINPHONI-2 is an open-label randomized trial (UMIN-CTR: UMIN000002730) that followed the Guidelines for Good Clinical Practice and adhered to the principles of the Declaration of Helsinki (2002) of the World Medical Association. The study protocol was approved by the Tohoku University Hospital Ethics Committee and the Institutional Ethics Committee at each site. Written informed consent was obtained from all patients or their representatives.

## Results

### Demographic data

**Figure 2:**
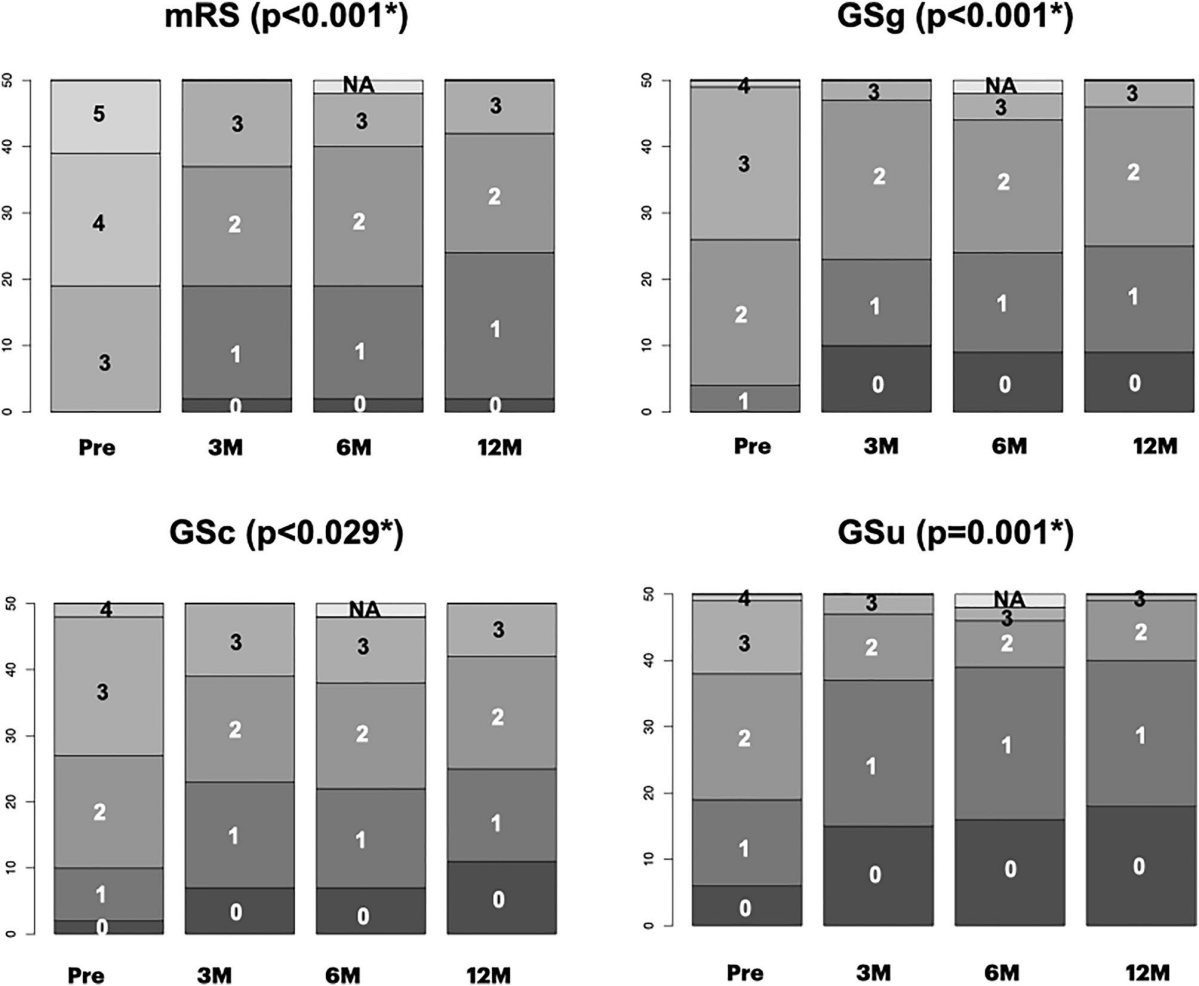
Sequential changes in general activity and NPH triad symptoms. In ADL assessed with mRS, lower grades (less disability) increased after surgery. All triad NPH symptoms assessed with the Japanese NPH grading scale-revised revealed statistically significant improvements. *Number* grades in respective grading systems, *ADL* activity of daily living, *GSg* grading scale of gait, cognition and urination in idiopathic normal pressure hydrocephalus, *Pre* preoperative state, *3M, 6M, 12M* 3, 6, and 12 months after surgery, *NA* not assessed.

Demographic data are shown in Table 1. In the IS group, data were obtained at the time of registration and 3, 6, and 12 months after surgery. In the PS group, preoperative data were collected 3 months after registration, immediately before shunt operation. The PS group exhibited lower mRS scores 3 months after registration than at registration, but the difference was not statistically significant. Postoperative data in the PS group were obtained similarly to those in the IS group. The preoperative differences between the IS and PS groups were not statistically significant, except for sex. Because our interest was focused on shunt responders, data from the IS and PS groups were combined for statistical analysis. Symptomatic improvements (improvement of one point or more on the iNPH grading scale) were observed in 70.0% of cases of gait, 60.4% of cognition, and 60.2% of urination 12 months operatively. Sequential changes in general activity and NPH symptoms assessed using the mRS and iNPH grading scales are shown in Fig. 2. All parameters showed significant improvement.

**Table 1:** Clinical characteristics of the participants.

	Total	IS group	PS group	p
Number of patients	50	30	20	(IS vs. PS)
Age (mean/SE)	76.2/0.7	76.2 /0.8	76.1/1.2	0.90
Sex, male (%)	56.0	36.7	85.0	< 0.01*
TUG time (mean/SE) (s)	30.7/5.2	34.0/43.9	25.1/6.1	0.38
MMSE total (mean/SE)	20.9/0.8	21.5/1.1	19.9/1.1	0.62
FAB total (mean/SE)	10.0/0.5	9.8 /0.6	10.4/0.9	0.31
Education (mean/SE) (years)	12.4 /0.5	12.2/0.6	12.8/0.2	0.56

Except for sex, no parameters, including the mRS score, showed statistically significant differences between the IS and PS groups. *IS* immediate surgery, *PS* postponed surgery, *p* probability, *q1* first quantile, *q3* third quantile, *FAB* Frontal Assessment Battery, *MMSE* minimal state examination, *mRS* modified Rankin scale, *TUG* Timed Up and Go test; {}, numbers in the PS group at registration.

### Sequential changes in TUG time and MMSE and FAB total scores

**Figure 3:**
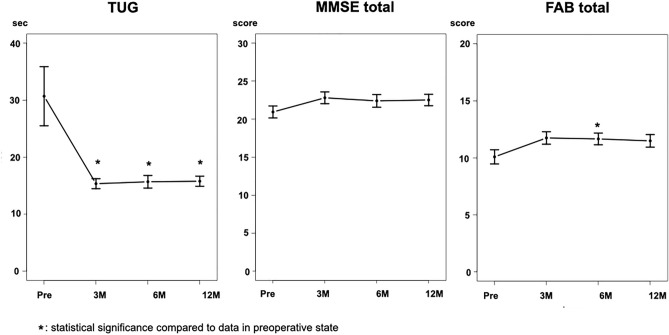
Sequential changes in TUG time and MMSE/FAB total scores. The TUG time shows a statistical improvement at 3, 6, and 12 months after surgery (3M, 6M, 12M). The MMSE total score increased after surgery, but no statistical significance is observed. The FAB total score shows a significant improvement at 6 months. *FAB total* total score of Frontal Assessment Battery, *MMSE* *total* total score of the mini-mental state examination, *TUG* Timed Up and Go test.

Sequential changes in the TUG time, MMSE score, and FAB total score as continuous variables are shown in Fig. 3. The TUG time decreased significantly at 3 months, and this improvement was almost maintained for up to 12 months. The total MMSE score increased at 3 months, and this increase was maintained for up to 12 months. However, this increase was small and insignificant. The FAB total score at 6 months showed a significant increase, and the data at 3 and 12 months also showed increases; however, these were not statistically significant.

### Sequential changes of the MMSE sub-item scores

Sequential changes in the frequencies of the MMSE sub-items at the respective levels are shown in Table 2. None of the sub-items were statistically significant. The scoring of the “Name” sub-item did not show any change, and all scores were 2 points (ceiling effect). Therefore, it was excluded from the subsequent analyses.

**Table 2:** Sequential changes in the frequency of the MMSE sub-item scores.

Level	Time: p = 0.689				Place: p = 0.233				Serial: p = 0.162			
	Pre	3M	6M	12M	Pre	3M	6M	12M	Pre	3M	6M	12M
“0”	3	2	5	3	2	2	4	4	10	4	4	5
“1”	3	1	1	2	5	2	0	1	14	18	14	18
“2”	6	3	3	2	7	2	2	2	8	5	10	6
“3”	4	10	5	8	6	8	6	9	8	4	0	6
“4”	13	11	16	15	10	10	11	10	6	7	3	2
“5”	21	23	18	20	20	26	25	24	4	12	12	13
NA	0	0	2	0	0	0	2	0	0	0	2	0

Level	Regist: p = 0.58				Recall: p = 0.07				3-step: p = 0.061			
	Pre	3M	6M	12M	Pre	3M	6M	12M	Pre	3M	6M	12M
“0”	1	1	0	0	21	15	12	12	2	0	0	0
“1”	0	0	1	1	8	13	9	12	0	1	1	1
“2”	2	2	1	2	15	7	10	13	10	2	4	6
“3”	47	47	46	47	6	15	17	13	38	47	43	43
NA	0	0	2	0	0	0	2	0	0	0	2	0

Level	Name: NA				Repeat: p = 0.275				Copy: p = 0.172			
	Pre	3M	6M	12M	Pre	3M	6M	12M	Pre	3M	6M	12M
“0”	1	0	0	1	12	8	8	11	16	14	11	12
“1”	0	0	0	0	38	42	40	39	34	36	37	38
“2”	49	50	48	49	–	–	–	–	–	–	–	–
NA	0	0	2	0	0	0	2	0	0	0	2	0

Level	Write: p = 0.315				Read: p = 0.059							
	Pre	3M	6M	12M	Pre	3M	6M	12M				
“0”	13	12	11	9	7	2	2	2				
“1”	37	38	37	41	43	48	46	48				
NA	0	0	2	0	0	0	2	0				

The frequencies of the levels of the respective sub-items were not statistically significant. On the score of the “Name” sub-item, all were “2” (ceiling effect). Therefore, it was excluded. *3M, 6M, 12M* 3, 6, and 12 months after surgery, *NA* not assessed, *p* probability.

### Relationship between TUG time and MMSE sub-items

**Figure 4:**
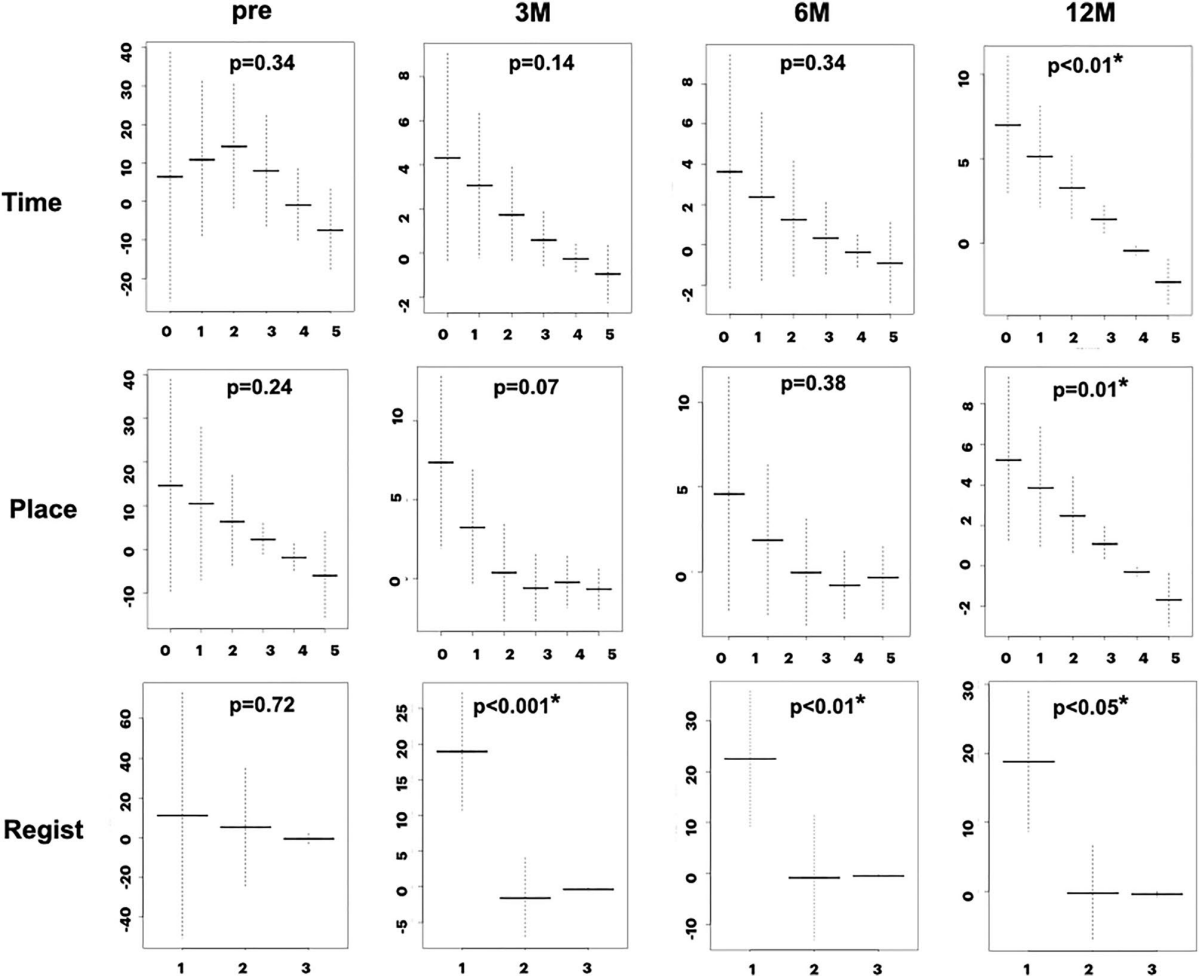
Association between TUG time and MMSE ordinal sub-items (Time, Place, Registration). The “Time” and “Place” sub-items shows a statistically significant association at 12 months. The “Registration” sub-item shows a significant association at 3, 6, and 12 months. *Y-axis* coefficients, *p* probability, *Pre* preoperative state, *3M, 6M, 12M*, 3, 6, and 12 months after surgery.

**Figure 5:**
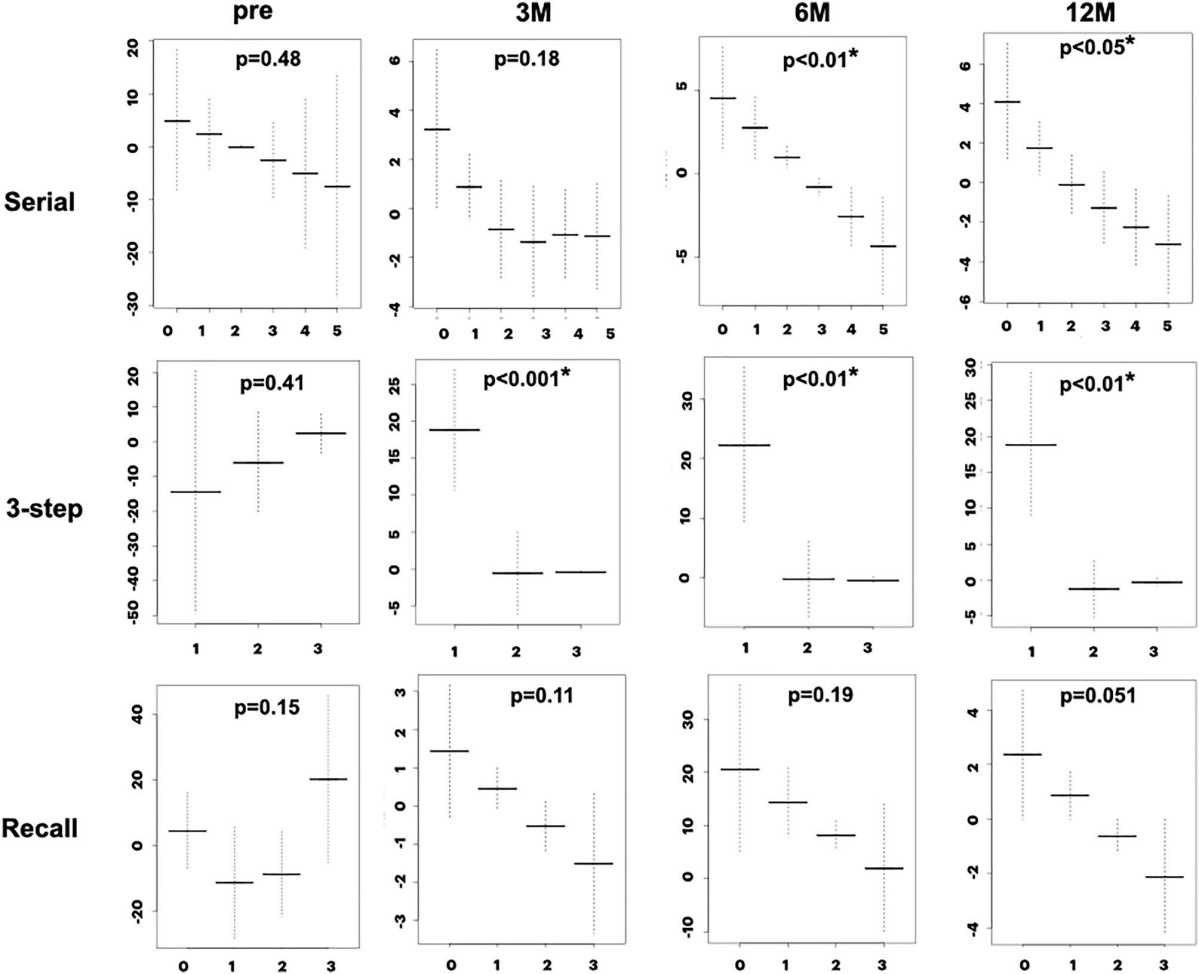
Association between TUG time and MMSE ordinal sub-items (Serial, 3-step commands, Recall). The “Serial 7” and “3-step command” sub-items show a significant association at 3, 6, and 12 months. Meanwhile, the “Recall” sub-item did not show an association after surgery. *Y-axis* coefficients, *Serial* serial 7 s calculation, *3-step* three-step command, *Recall* delayed recall of three words, *p* probability, *Pre* preoperative state, *3M, 6M, 12M*, 3, 6, and 12 months after surgery.

**Figure 6:**
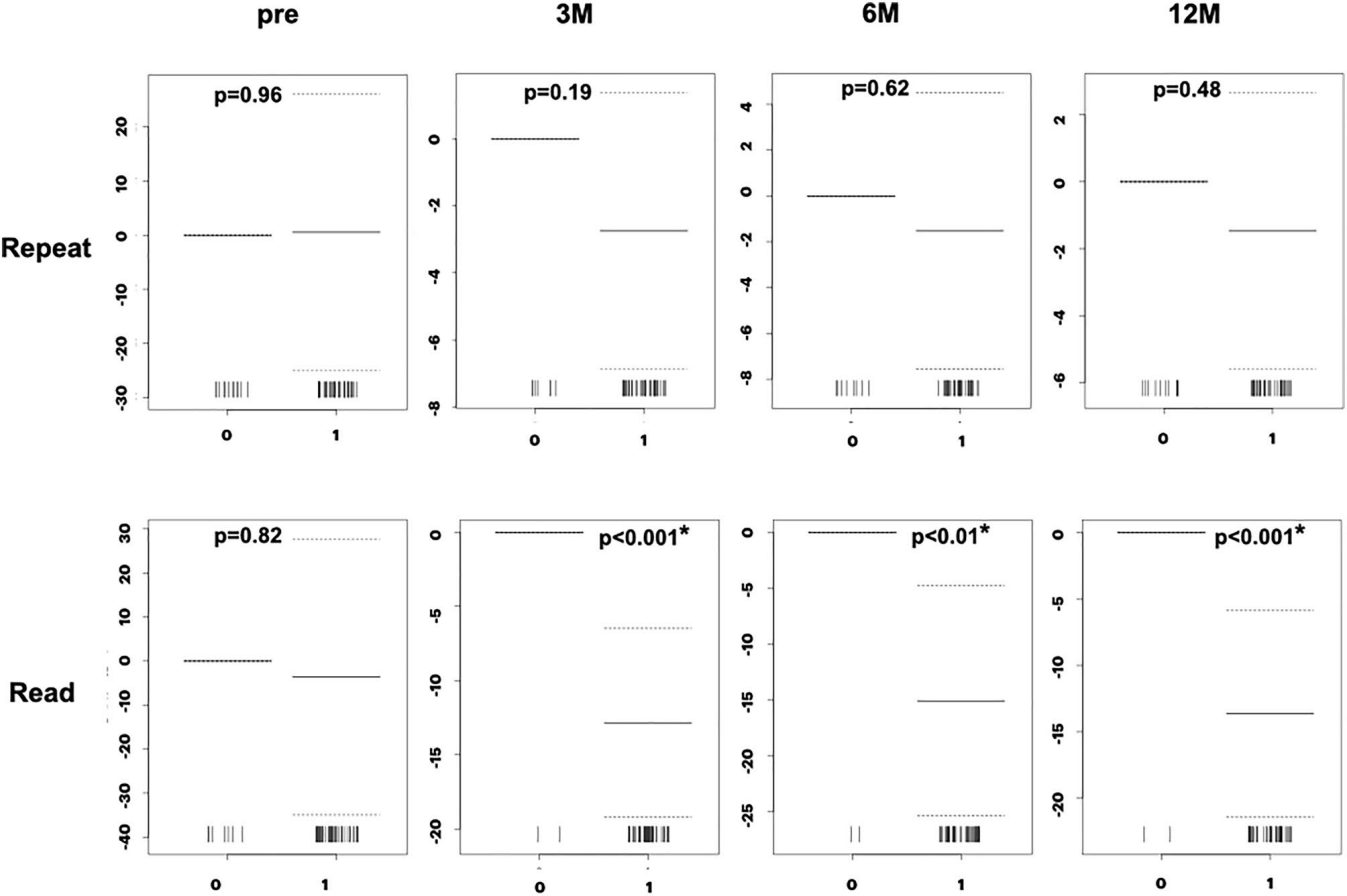
Association between TUG time and MMSE binary sub-items (Repeat, Read). The “Repeat” sub-item did not show an association after surgery. The “Read” sub-item shows a significant association at 3, 6, and 12 months. *Y-axis* coefficients, *Read* read and obey, *Repeat* repeat the phrase, *p* probability, *Pre* preoperative state, *3M, 6M, 12M* 3, 6, and 12 months after surgery.

**Figure 7:**
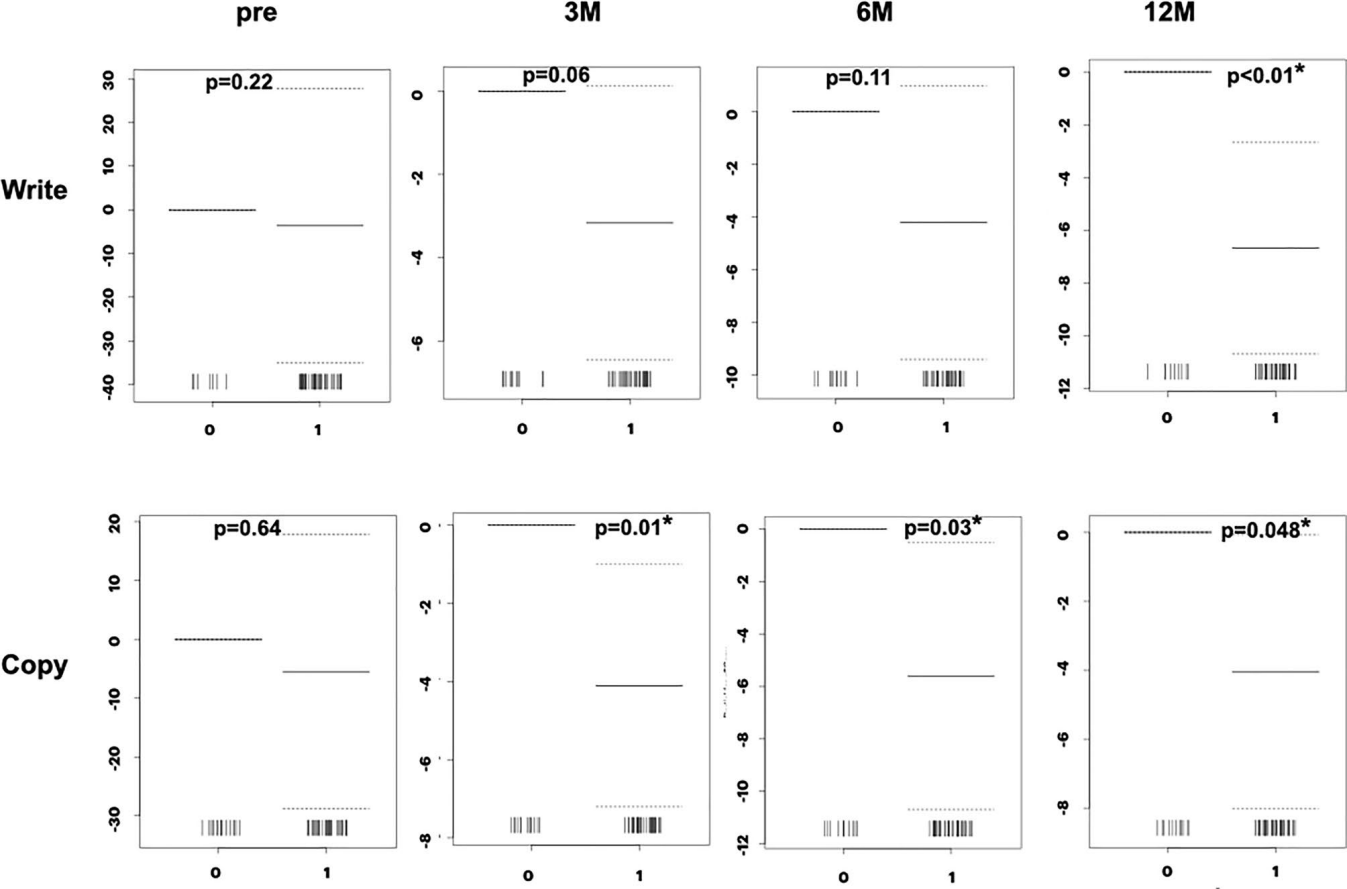
Association between TUG time and MMSE binary sub-items (Write, Copy). The “Write” sub-item shows the association at 12 months. The “Copy” sub-item shows a significant association at 3, 6, and 12 months. Y-axis, coefficients; p, probability; Pre, preoperative state; 3M, 6M, 12M, 3, 6, and 12 months after surgery.

The relationship between TUG time and MMSE sub-items was examined for ordinal and binary variables. Sequential changes in the mean and standard errors of the ordinal sub-items are plotted in Figs. 4 and 5. The sequential changes in the binary sub-items are plotted in Figs. 6 and 7. The y-axis shows the coefficients of the ordinal predictors with TUG time, and the x-axis shows each ordinal predictor level. Because the TUG time is inversely related to gait speed, lower coefficients for higher levels of ordinal predictors indicate a close relationship between gait and cognition. Sequential changes in the approximate significance of the smoothing terms and parametric coefficients using the ordinal smoothing penalty and GAM are presented in Table 3.

**Table 3:** Sequential changes in the relationship between TUG and MMSE sub-items.

	Pre	3M	6M	12M
N		48	47	48
Ordinal	Edf (dev.expl), p	Edf (dev.expl), p	Edf (dev.expl), p	Edf (dev.expl), p
Time	1.88 (7.86%), 0.34	1.30 (7.76%), 0.14	1.20 (3.82%), 0.34	1 (20.1%), < 0.01*
Place	1 (2.89%), 0.24	2.31 (16.8%), 0.07	1.65 (5.52%), 0.38	1 (12.5%), 0.01*
Regist	1 (0.27%), 0.72	1.91 (33.3%), < 0.001*	1.76 (21.8%), < 0.01*	1.84 (21.8%), < 0.01*
Serial	1 (1.06%), 0.48	1.96 (11%), 0.18	1 (16.1%), < 0.01*	1.57 (18.3%), < 0.01*
3-step	1 (1.41%), 0.41	1.88 (32.8%), < 0.001*	1.86 (21.6%), < 0.01*	1.91 (24.9%), < 0.01*
Recall	2.1 (17.5%), 0.15	1.00 (5.57%), 0.11	1 (3.73%), 0.19	1 (8%), 0.051
Binary	Est (SE), p	Est (SE), p	Est (SE), p	Est (SE), p
Repeat	0.56 (12.73), 0.96	− 2.75 (2.06), 0.19	− 1.52 (3.01), 0.62	1.47 (2.06), 0.48
Read	− 3.62 (15.66), 0.82	12.84 (3.18), < 0.001*	− 15.08 (5.15), < 0.01*	− 13.63 (3.87), < 0.001*
Write	− 15.05 (12.20), 0.22	− 3.17 (1.64), 0.06	− 4.21 (2.60), 0.11	− 6.67 (2.00), < 0.01*
Copy	− 5.51 (11.63), 0.64	− 4.10 (1.56), 0.01*	− 5.60 (2.54), 0.03*	− 4.04 (1.99), 0.048*

The number of statistically significant sub-items (with *) increased during the follow-up period (3M, 6M, 12M: 3, 6, and 12 months after surgery). The effective degrees of freedom (Edf) show that 1 is equivalent to linearity and edf > 2 is highly non-linear. *Est* estimate, *dev.expl* deviance explained, *p* probability, *SE* standard error, *TUG* Timed Up and Go test, *MMSE* Mini-Mental State Examination.

As shown in Figs. 4 to 7 and Table 3, the ordinal and binary sub-items in the preoperative state did not show a statistically significant association with TUG time. At 3 months after surgery, the “Regist,” ”3-step,” “Read,” and “Copy” sub-items showed a statistically significant association with gait. At 6 months, the “Serial” sub-item showed a significant association, in addition to the “Regist,” ”3-step,” “Read, and “Copy” sub-items. At 12 months, the “Time,” “Place,” and “Write” sub-items showed significant association, in addition to the “Regist,” ”3-step,” “Read,” “Copy,” and “Serial” sub-items. The “Recall” sub-item at 12 months showed a p-value of 0.051, close to the cut-off value for significance. Therefore, almost all sub-items showed a statistically significant association with the TUG time. The number of sub-items with statistical significance increased during follow-up. Only the “Repeat” sub-item did not show a significant association.

## Discussion

This study investigated the association between gait and cognition in patients with iNPH using the TUG time and the MMSE sub-items. The MMSE sub-items were treated as ordinal, neither continuous nor categorical, using a newly developed statistical method. Preoperatively, no sub-items showed an association with gait. In contrast, at 3 months after surgery, the “Regist,” ”3-step,” “Read,” and “Copy” sub-items showed a statistically significant association, and the number of statistically significant sub-items increased during follow-up. This study revealed that the association between gait and MMSE sub-items gradually increased in patients with iNPH after surgery.

First, the combination of IS and PS data used in this study is discussed. The preoperative state did not differ significantly between the two groups, as shown in Table 1. The PS group showed a slightly lower score on mRS 3 months after registration than at registration, but there were no statistical differences between them. Furthermore, in the SINPHONI-2, there was no statistical difference between the two groups at 1 year postoperatively^11,12^. This indicates that a 3-month delay in surgery can reverse symptoms to almost the same degree as in the IS group. Early treatment of patients with iNPH has increased survival compared to delayed surgery^25^. However, the delayed surgery group in this report waited for surgery for 6.8–23.8 months (median, 13.2 months). This delay was longer than that of SINPHONI-2. Regarding the waiting time for surgery in patients with iNPH, the outcomes were compared between the three groups of < 3 months, < 6 months, and more^26^. The highest outcome was observed at 12 months in the group with a waiting time of < 3 months. Therefore, early surgery is preferable for < 3 months after surgical recommendation.

iNPH in older adults has become well recognized since the publication of the iNPH clinical guidelines^2,27^. Although iNPH is a well-known and treatable form of dementia^1^, it is unclear whether there are improvements in cognitive disturbances. Our previous study on caregiver burden using SINPHONI-2 data revealed improvements in cognition after surgery, but not to the same degree as gait^3^. In contrast, a recent study reported that most patients (77%) with iNPH improved and maintained cognitive function for at least 2 years after surgery^28^. A systematic review revealed that cognitive improvement after surgery was observed only in some limited measures, including the MMSE, Rey Auditory Verbal Learning Test, and Trail Making Test A^29^.

Recently, the association between gait and cognition has become an important topic. An association between gait and cognition has been reported in community-dwelling older adults^9^ and older adults with mild cognitive impairment and Alzheimer’s dementia^30^. An association between frontal lobe dysfunction and gait disturbances has been reported in patients with iNPH^10^. In this study, the MMSE total scores did not correlate significantly with the TUG time. In contrast, the increase in the FAB score was reported to be significant at 6 months and close to being significant at 3 and 12 months. The FAB was designed to evaluate frontal executive function. The increase in total FAB scores observed in our study indicated that frontal lobe function and gait improved after surgery in patients with iNPH.

The present study investigated the clinical significance of the MMSE sub-items using an ordinal smoothing penalty with GAM. The ordinal smoothing penalty increases the reliability of the assessment of ordinal predictors^7,8^. GAM allows statistical inference of ordinal predictors^7^. The GAM is an extension of the commonly used generalized linear mixed model, which allows modeling of nonlinear predictors while maintaining interpretability^23^. GAM also allows for statistical inference of continuous predictors. The use of this combination is promising for various research fields that involve ordinal predictors.

In this study, the newly developed statistical method shows that “Regist,” “3-step,” “Read,” and “Copy” showed significant associations 3 months after surgery. This suggests that the immediate memory, attention, and visuoconstructive domains improved in the early postoperative period. The association between gait and the MMSE score increased with time. This association delays orientation to time and place. This study indicates that the association between gait and sub-items gradually increased after surgery in patients with iNPH. These findings were not consistent with the sequential changes in the sub-items that did not show any significant changes, as shown in Table 2. The latter only showed sequential changes in the frequency of MMSE sub-item scores, not associated with TUG time.

This study had several limitations and strengths. First, the sample size is small. As this study aimed to investigate the relationship between gait and cognition in patients with definite iNPH, we focused on shunt responders 12 months after surgery. Therefore, the number of patients in the present study decreased from 83 to 50. Second, the scores on several MMSE sub-items are only one or two points, and they can show a ceiling effect with no variance among patients. In our study, the “Name” sub-item showed a ceiling effect, making accurate data interpretation difficult. A major strength of this study is the potential for examining MMSE sub-item scores as ordinal and binary variables using a newly developed statistical method. They can be applied to various fields of research. Second, this is the first study of iNPH to investigate the association between gait and cognition.

## Conclusions

The association between gait and cognition, as assessed by the MMSE sub-items, was observed in patients with iNPH after shunt surgery. This study shows the potential use of MMSE sub-items to study cognitive changes in various cognitive fields. The newly developed statistical method, which uses the ordinal smoothing penalty with GAM, is useful for assessing data on an ordinal scale.

### Supplementary Information

Supplementary Information.

## Data Availability

The datasets generated and analyzed in this study are included in this published article and its Supplementary Information file.
